# Maresin 1 Attenuates Radicular Pain Through the Inhibition of NLRP3 Inflammasome-Induced Pyroptosis via NF-κB Signaling

**DOI:** 10.3389/fnins.2020.00831

**Published:** 2020-08-26

**Authors:** Yi-hao Wang, Yan Li, Jun-nan Wang, Qing-xiang Zhao, Jin Jin, Shuang Wen, Si-cong Wang, Tao Sun

**Affiliations:** ^1^Department of Pain Management, Shandong Provincial Hospital, Cheeloo College of Medicine, Shandong University, Jinan, China; ^2^Department of Anesthesiology, Qingdao Municipal Hospital, Qingdao, China; ^3^Department of Obstetrics and Gynecology, The Affiliated Hospital of Qingdao University, Qingdao, China

**Keywords:** radicular pain, maresin 1, NLRP3 inflammasome, pyroptosis, NF-κB

## Abstract

**Background:**

The exposure of the nucleus pulposus (NP) causes an immune and inflammatory response, which is intrinsically linked to the pathogenesis of radicular pain. As a newly discovered pro-resolving lipid mediator, maresin 1 (MaR1) could exert powerful inflammatory resolution, neuroprotection, and analgesic activities. In the present research, the analgesic effect of MaR1 was observed. Then, the potential mechanism by which MaR1 attenuated radicular pain was also analyzed in a rat model.

**Methods:**

Intrathecal administration of MaR1 (10 or 100 ng) was successively performed in a rat with non-compressive lumbar disk herniation for three postoperative days. Mechanical and thermal thresholds were determined to assess pain-related behavior from days 1 to 7 (*n* = 8/group). On day 7, the tissues of spinal dorsal horns from different groups were gathered to evaluate expression levels of inflammatory cytokines (IL-1β, IL-18, and TNF-α), the NLRP3 inflammasome and pyroptosis indicators (GSDMD, ASC, NLRP3, and Caspase-1), together with NF-κB/p65 activation (*n* = 6/group). TUNEL and PI staining were performed to further examine the process of pyroptosis.

**Results:**

After intrathecal administration in the rat model, MaR1 exhibited potent analgesic effect dose-dependently. MaR1 significantly prompted the resolution of the increased inflammatory cytokine levels, reversed the up-regulated expression of the inflammasome and pyroptosis indicators, and reduced the cell death and the positive activation of NF-κB/p65 resulting from the NP application on the L5 dorsal root ganglion.

**Conclusion:**

This study indicated that the activation of NLRP3 inflammasome and pyroptosis played a significant role in the inflammatory reaction of radicular pain. Also, MaR1 could effectively down-regulate the inflammatory response and attenuate pain by inhibiting NLRP3 inflammasome-induced pyroptosis via NF-κB signaling.

## Introduction

Radicular pain has become a public health concern with increasing prevalence and associated disability (Manchikanti and Hirsch, 2015), and lumbar disc herniation (LDH) is the most commonly identified contributor (Valat et al., 2010). There is increasing recognition that neuroinflammation caused by exposure of the nucleus pulposus (NP) is intrinsically linked to the pathogenesis of radicular pain (Geiss et al., 2007; Park et al., 2011; Di Martino et al., 2013). The avascular NP fails to acquire immunological tolerance under normal conditions (Geiss et al., 2009), so its exposure to the immune system causes an intense autoimmune and inflammatory cascade in the nervous system, which results in central sensitization and unbearable pain (Valat et al., 2010; Zhu et al., 2014). Proinflammatory cytokine IL-1β plays a crucial function in the development of inflammatory pain (Nadeau et al., 2011; Yano et al., 2013).

The NOD-like receptor (NLR) family pyrin domain-containing protein 3 (NLRP3) inflammasome is an important molecular platform (Lamkanfi and Dixit, 2014), which can be activated by endogenous and exogenous dangerous signals to contribute to releasing IL-18 and IL-1β and promoting their maturation (Nakahira et al., 2011; Walsh et al., 2014; Moon et al., 2016). Simultaneously, activated inflammasome triggers pyroptosis, a newly discovered proinflammatory programmed cell death, which cleaves cell membrane and induces extravasation of cellular contents, leading to inflammatory cascades (Shi et al., 2015). Several studies have shown that NLRP3 inflammasome activation contributed to the development of neuropathic pain (Jia et al., 2017; Jin et al., 2017; Pan et al., 2018). Meanwhile, pyroptosis was involved in the neuroinflammation induced by brain injury or multiple sclerosis (Liu et al., 2018; McKenzie et al., 2018). Therefore, the regulation of pyroptosis mediated by inflammasome is expected to be an ideal strategy for the therapy of neuroinflammatory pain.

It has been demonstrated that the inflammation resolution is regulated by endogenous specialized pro-resolving mediators (SPMs), e.g., lipoxins, resolvins, maresins, and protectins, indicating that it is an active, programmed process (Buckley et al., 2014; Serhan, 2014). Lipoxins and resolvins could effectively inhibit mechanical hypersensitivity and promote inflammatory resolution in our previous studies (Miao et al., 2015; Liu et al., 2016; Zhang et al., 2018). Maresins, a newly discovered pro-resolving family of docosahexaenoic acid (DHA)-derived mediators from macrophages, also exert pro-resolving and anti-inflammatory (Serhan et al., 2009), neuroprotective (Francos-Quijorna et al., 2017), and analgesic activities (Serhan et al., 2012) to maintain host homeostasis.

Pro-resolving lipid mediators could effectively suppress the immune response by repressing the activation of NLRP3 inflammasome (Lopategi et al., 2019). In addition, the nuclear factor-κB (NF-κB)/p65 signal functions in the generation of neuropathic pain (Sun et al., 2012) and inflammasome activation (Zhang et al., 2017). Also, Resolvin D1 relieved sciatica by modulating the expression of NF-κB (Liu et al., 2016). However, few studies have evaluated the role of SPMs in the process of pyroptosis, especially in models of neuropathic pain. We hypothesized that the exposure of NP activated inflammasome as an endogenous dangerous signal, which further triggered pyroptosis to exacerbate the immune response in radicular pain, and that maresin 1 (MaR1) could regulate pyroptosis to inhibit the immune and inflammatory responses via the NF-κB pathway.

## Materials and Methods

### Experimental Animals

According to the International Association for the Study of Pain’s laboratory animal guidelines, Shandong University Animal Care and Use Committee approved this animal experiment. Rat models in this research were adult Sprague–Dawley rats (male, 220–260 g), which were supplied by Shandong University Experimental Animal Centre (Shandong, China). We separated the rats in groups (3–5 rats per cage). Each group was provided standard rodent chow and water and raised with a light/dark cycle of 12 h. The temperature was constantly maintained at 25 ± 1°C, and the humidity was in a comfortable range of 45 ± 5%.

### Disc Herniation Model

The protocol for non-compressive lumbar disc herniation (NCLDH) models was performed as previously described in detail (Kim et al., 2011; You et al., 2013). The rats were anesthetized by being intraperitoneally injected with 50 mg/kg sodium pentobarbital. Then, over the lumbar spine, an incision was cut following the dorsal midline to separate the multifidus muscles following the L4–L6 spinous processes. Laminectomy was used to expose the dorsal root ganglion (DRG) as well as the right L5 spinal nerve root. The equivalent NP (about 5 mg) that was from two coccygeal intervertebral disks was placed on L5 DRG. It should be noted that the mechanical compression should be carefully minimized. The surgical process for the control group was consistent, including the extraction of NP, except for the application of the NP. All surgical procedures were performed under sterile conditions.

### Intrathecal Catheterization

Once the model was successfully finished, the intrathecal injection was performed by embedding polyethylene catheters (PE-10; Smiths Medical, United Kingdom). The protocol was followed as previously described (Choi et al., 2005). From the L5–L6 intervertebral foramen, the intrathecal space was embedded with a PE-10 catheter until the cerebral spinal fluid outflowed. After being effectively fixed under the skin, the external catheter was sutured firmly at the head. Intrathecal catheterization was verified to be successful if the lower limbs of a rat dragged or were paralyzed by intrathecally injecting 2% lidocaine (10 μl) after recovery from anaesthesia.

### Chemical Administration

MaR1 was obtained from Cayman Chemical Company (United States). Firstly, the reagent was sub-packed in EP tubes for separated rats. Following the manufacturer instructions, a gentle stream of nitrogen was used to evaporate the MaR1 from the solvent ethanol and then redissolved immediately in sterile phosphate-buffered saline (PBS). After being prepared, MaR1 was given to rats within 15 min. With a 10 μl microinjection syringe connected to the intrathecal catheter, MaR1 (10 or 100 ng, 10 μl) or vehicle (PBS, 10 μl) was administered to rats via intrathecal injection. The administration was conducted successively for the first 3 days after the operation. The dose of MaR1 was determined in accordance to Francos-Quijorna’s study (Francos-Quijorna et al., 2017), and we made a conversion in accordance with the weight and injective route.

### Assessment of Pain-Related Behavior

Assessment of paw withdrawal latency (PWL) to thermal stimulus and that of paw withdrawal thresholds (PWTs) to mechanical stimulus were performed to evaluate the pain-related behavior, as described previously (Hargreaves et al., 1988; Chaplan et al., 1994). The concrete methods were performed according to the detailed description (Pan et al., 2018). The tests were conducted 1 day before the surgery, while successively from the 1st day to the 7th day after surgery. Before the test, rats were individually placed in the testing environment for 1 h for acclimation. The investigator was blinded to the medication of rats. The mechanical thresholds (PWTs) in each hind paw were assessed by using Von Frey filaments (1–10 g, Stoelting, United States) following the “up–down” approach. The degree of PWL, a measure of thermal thresholds, was measured using Hargreaves’ test (Ugo Basile, Varese, Italy). For both methods, we counted the mean value of five measurements for each animal per test section with intervals of 5 min or more.

### Tissue Collection for ELISA, PCR, and Western Blot Analysis

The ipsilateral spinal dorsal horn tissue from lumbar enlargement was harvested on the 7th postoperative day (*n* = 6/group). Before tissue collection, the Sprague–Dawley rats were perfused fully with normal saline via the heart until their livers became white. All of these tissues were preserved in liquid nitrogen.

### Reverse Transcription–Polymerase Chain Reaction

Complying with the manufacturer instructions, TRIzol reagent (Life Technologies, United States) was employed to extract the total RNA of ipsilateral spinal dorsal horn tissues. NanoDrop 2000 (Thermo Fisher Scientific, United States) was utilized to detect the RNA purity and concentration. Then, the PrimeScript RT reagent kit (Takara, Dalian, China) was used to reversely transcribe the RNA. SYBR Green Premix Ex Taq (TaKaRa, Dalian, China) was used for performing the polymerase chain reaction (PCR) amplifications, and a Light Cycler^®^ 480 II (Roche, Switzerland) was employed to perform the reactions. Through the normalization of the levels of mRNA in relation to that of β-actin, the 2-ΔΔCt method was applied to perform the analysis of mRNA. The synthesis of the primers was conducted by BioSune Company (China); the sequences were presented in Table 1.

**TABLE 1:** The Primer Sequences for RT-PCR.

Gene	Length	Primer	Sequences
NLRP3	291 bp	Forward	5′ CTGCATGCCGTATCTGGTTG 3′
		Reverse	5′ CGGCGTTAGCAGAAATCCAG 3′
ASC	188 bp	Forward	5′ CCATCCTGGACGCTCTTGAAA 3′
		Reverse	5′ TGTGAGCTCCAAGCCATACC 3′
Caspase-1	137 bp	Forward	5′ GAACAAAGAAGGTGGCGCAT 3′
		Reverse	5′ CAAGACGTGTACGAGTGGGT 3′
GSDMD	171 bp	Forward	5′ CTGACTCTTCGAGAACCGCT 3′
		Reverse	5′ CTGACGGCATGATCCACGAT 3′
β-actin	138 bp	Forward	5′ TTACTGCCCTGGCTCCTAG 3′
		Reverse	5′ CGTACTCCTGCTTGCTGATC 3′

### Enzyme-Linked Immunosorbent Assay

To assess the inflammatory cytokines’ protein levels in the spinal dorsal horns, we used enzyme-linked immunosorbent assay (ELISA) kits (RD Systems, United States; ExCell, China). The tissues were harvested from the rats, followed by homogenization and centrifugation, as previously described (Liu et al., 2016). The supernatants were assayed following the instructions of ELISA kits.

### Western Blot Analysis

Protein from tissues of spinal dorsal horn was extracted in RIPA lysis buffer, which was bought from Beyotime Biotechnology of China. Through BCA Protein Assay (Thermo Fisher Scientific), the protein concentration was assessed. To separate 30 μg of protein samples, sodium dodecyl sulfate–polyacrylamide gel electrophoresis (SDS-PAGE) was then utilized. After separation, protein samples were placed in a polyvinylidene difluoride (PVDF) nitrocellulose membrane (Millipore, Boston, MA, United States), arrested by 5% fat-free dry milk at 25°C in TBST for 1 h and cultured at 4°C with primary antibodies overnight. Primary antibodies included the following: anti-ASC (1:500; ABclonal), anti-Nlrp3 (1:1000; Proteintech), anti-GSDMD (1:1000; Abcam), anti-cleaved Caspase-1 (1:1000; Proteintech), anti- NF-κB/p65 (1:2000; Abcam), and anti-GAPDH (1:10,000; Cell Signaling Technology, CST). The membranes were cultured with a rabbit or mouse HRP-conjugated secondary antibody (1:2000; Beyotime Biotechnology) at 25°C for 1 h after being washed in TBST. Finally, the densities of the target proteins relative to that of GAPDH were quantified using Image Pro Plus software.

### Immunohistochemistry

Immunohistochemistry was performed according to a previous description (Liu et al., 2016). A series of spinal cord tissue processing steps were conducted, such as perfusion, fixation, embedding, dewaxing, rehydration, deparaffinization, and antigen retrieval. At 4°C, the sections were then cultured with anti-GSDMD antibody (1:1000; Abcam), anti-Nlrp3 (1:1000; Proteintech), and anti-NF-κB/p65 antibody (1:500; Abcam) overnight. Thereafter, the incubation of the sections with a secondary antibody was maintained at 37°C for 30 min. After staining, an Olympus BX60 microscope (Olympus Optical Co., Ltd., Tokyo, Japan) was applied to acquire images of the specimens.

### Analysis of Cell Death

On the 7th day, the spinal cord tissues were collected and cryo-preserved at −80°C from different groups. For PI staining, the PI dye (1 mg/kg, 100 μl) was perfused intraperitoneally 1 h before the rats were killed. The frozen spinal cord sections (7 μm) were fixed in ice-cold acetone for 10 min. 0.1% Triton X-100 was added for 30 min before blocking with 5% FBS under shade for 1 h. Then, the sections were incubated with primary antibody (anti-Caspase-1, 1:100) at 4°C overnight. Following washing with PBST [0.5% Tween-20 with phosphate-buffered saline (PBS)] three times, proper secondary antibodies (Dylight 488, 1:200, Abcam, United States) were added to further incubate for 2 h at room temperature. For TUNEL staining, the sections were incubated with TUNEL dye (Roche, United States) for 30 min under shade. After being washed with TBST, the samples were counterstained with DAPI (1:1000, Sigma, United States) for 5 min. The sections were imaged under a fluorescence microscope (OLYMPUS, Japan).

### Statistical Analysis

In the present study, all the results were given as the mean ± SD. Through one-way analysis of variance (ANOVA), we compared the differences between study groups, while for assessment of pain behaviors, we performed two-way ANOVA. Then, Bonferroni *post hoc* test was performed. 0.05 was the threshold of a two-sided *P*-value. SPSS 23.0 software (IBM, United States) was applied to perform the analysis, which was then illustrated by using GraphPad Prism^®^ 7.04 (GraphPad Software, United States).

## Results

### MaR1 Alleviated Mechanical Allodynia Induced by Non-compressive LDH

**FIGURE 1:**
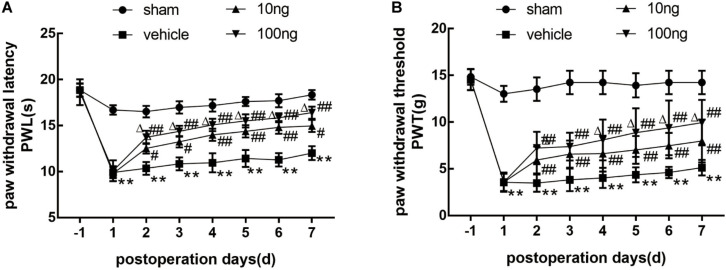
Application of MaR1 regulated pain behavior in a rat model of NCLDH (*n* = 8/group). **(A,B)** MaR1 (10 or 100 ng) reversed PWL and PWT induced by NCLDH from the 2nd day to the 7th postoperative day. Data are presented as the mean ± SD (**P < 0.01 versus the sham group; ^##^*P* < 0.01 versus the vehicle group; ^#^*P* < 0.05 versus the vehicle group; ^Δ^*P* < 0.05 versus the 10 ng MaR1 group).

In order to investigate whether MaR1 contributed to the alleviation of neuropathic pain, we first assessed the pain behavior in a rat model of NCLDH. The results (Figures 1A,B) indicated that in comparison with the sham group, both PWL and PWTs in the vehicle group (*n* = 8) dropped remarkably from the 1st day to the 7th day (*P* < 0.001 on each day). Thermal hyperalgesia as well as mechanical allodynia in the vehicle group was considerably reversed after the administration of MaR1 (10 or 100 ng) from the 2nd day to the 7th day (*P* < 0.05 on each day). In the 100 ng MaR1 group, the thermal allodynia was mitigated more significantly than in the 10 ng MaR1 group through the observation days (*P* < 0.05 on each day), while the mechanical allodynia was alleviated more markedly only on 4th day to 7th day (*P* < 0.05 on each day).

In addition, the contralateral PWL and PWTs did not show any differences among the four groups (Supplementary Figures S1A,B). Then, to demonstrate that the administration of MaR1 to sham groups did not induce motor deficits, we found that the mechanical and thermal hypersensitivity were not different in the sham rats with or without MaR1 treatment (Supplementary Figures S1C,D).

### MaR1 Attenuated the Proinflammatory Cytokines’ Expression

**FIGURE 2:**
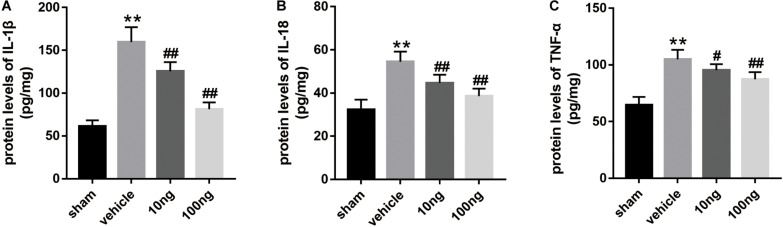
MaR1 decreased the spinal cord’s proinflammatory protein levels (*n* = 6/group). ELISA was employed to measure the inflammatory cytokines’ protein levels. **(A–C)** MaR1 (10 or 100 ng) reversed the rising protein levels of IL-1β, IL-18, and TNF-α induced by NCLDH. Data are presented as the mean ± SD (**P < 0.01 versus the sham group; ^##^*P* < 0.001 versus the vehicle group; ^#^*P* < 0.05 versus the vehicle group).

Our previous studies (Miao et al., 2015; Liu et al., 2016; Zhang et al., 2018) showed that SPMs inhibited mechanical hypersensitivity by regulating the levels of inflammatory cytokines. Therefore, ELISA was performed to evaluate whether MaR1 regulated the expression of IL-1β, IL-18, and TNF-α in ipsilateral spinal dorsal horns. As shown in Figures 2A–C, in comparison with the sham group, their protein levels markedly increased in the vehicle group (*P* < 0.001 for each cytokine). The dose-dependently intrathecal delivery of MaR1 (10 or 100 ng) significantly promoted the resolution of IL-1β (*P* = 0.001, *P* < 0.001), IL-18 (*P* = 0.001, *P* < 0.001), and TNF-α levels (*P* = 0.019, *P* < 0.001), compared with those in the vehicle group.

### Activation of NLRP3 Inflammasome and Pyroptosis in an NCLDH Rat Model

**FIGURE 3:**
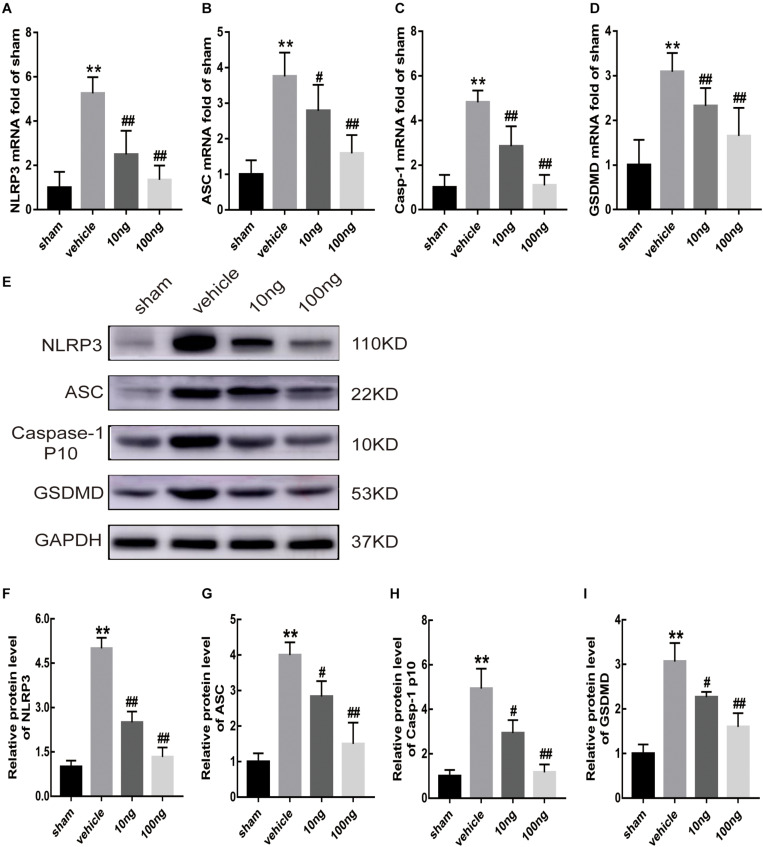
MaR1 inhibited the mRNA and protein expressions of inflammasome and pyroptosis indicators in spinal cord (*n* = 6/group). The inflammasome and pyroptosis indicators’ expression levels were determined by qRT-PCR and Western blot analysis. **(A–D)** MaR1 (10 or 100 ng) reversed the rising mRNA levels of NLRP3, ASC, Caspase-1, and GSDMD induced by NCLDH. **(E–I)** MaR1 (10 or 100 ng) reversed the increased protein levels of NLRP3, ASC, Caspase-1, and GSDMD induced by NCLDH. Data are presented as the mean ± SD (**P < 0.01 versus the sham group; ^##^*P* < 0.01 versus the vehicle group; ^#^*P* < 0.05 versus the vehicle group).

The secretion and maturation of two proinflammatory cytokines IL-18 and IL-1β (Nakahira et al., 2011; Moon et al., 2016) can be promoted by NLRP3 inflammasome activation, which could also trigger pyroptosis (Shi et al., 2015), resulting in the expansion of the inflammatory response. The qRT-PCR results illustrated that the mRNA expression of the inflammasome indicators (NLRP3, *P* < 0.001; ASC, *P* < 0.001; Caspase-1, *P* < 0.001) was up-regulated in ipsilateral spinal dorsal horns after NP application (Figures 3A–C). Interestingly, Figure 3D showed a more significant increase of the mRNA expression of gasdermin D (GSDMD), which is the crucial protein in the process of pyroptosis (Shi et al., 2015), in the vehicle group (*P* < 0.001).

Western blot analysis was performed to assess inflammasome and pyroptosis related indicators’ protein expression levels in ipsilateral spinal dorsal horns (Figures 3D–H). In comparison with the sham group, the protein expression levels of these indicators, including NLRP3, ASC, cleaved Caspase-1, and GSDMD (*P* < 0.01 for each indicator), were significantly higher after NP was applied to the L5 DRG.

**FIGURE 4:**
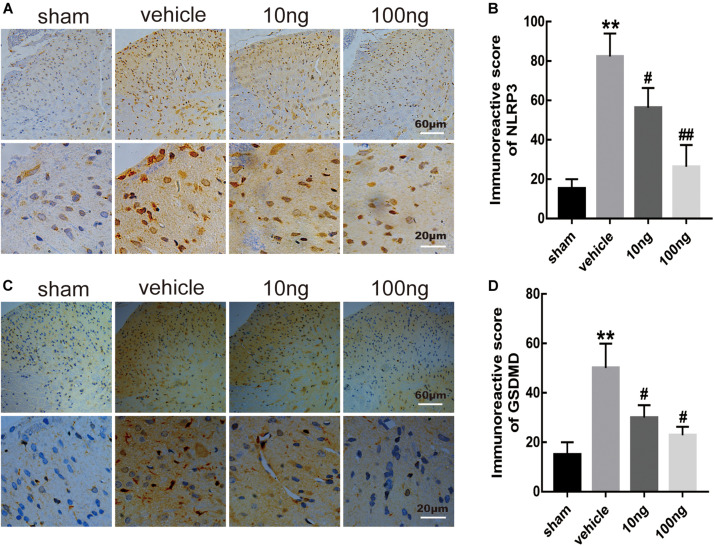
MaR1 inhibited the level of positive images of NLRP3 and GSDMD in spinal dorsal horns (*n* = 6/group). Immunohistochemistry was used to determine the levels of positive expression of NLRP3 and GSDMD. **(A–D)** MaR1 (10 or 100 ng) reversed the rising positive expression levels of NLRP3 and GSDMD induced by NCLDH. Data are presented as the mean ± SD (**P < 0.01 versus the sham group; ^##^*P* < 0.01 versus the vehicle group; ^#^*P* < 0.05 versus the vehicle group).

Moreover, to determine positive expression levels of the mentioned indicators in spinal dorsal horns, immunohistochemistry was conducted. Significantly, increased levels of positive expression of NLRP3 (*P* < 0.001) and GSDMD (*P* < 0.001) were exhibited in the vehicle group (Figures 4A–D).

**FIGURE 5:**
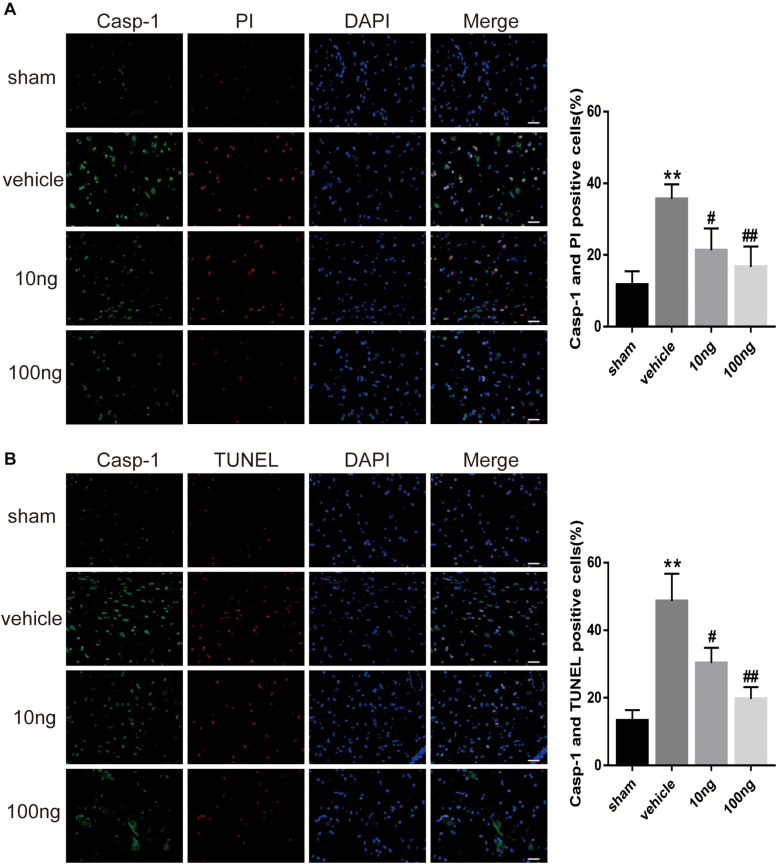
MaR1 attenuated pyroptosis in spinal dorsal horns (*n* = 5/group). Double immunofluorescence staining was employed to examine the pyroptosis in spinal dorsal horns. It was determined as pyroptosis if double positive of cleaved Caspase-1 and TUNEL (or PI) occurred. **(A,B)** The frequency of pyroptotic in spinal cord was attenuated after the application of MaR1 dose-dependently. Data are given as the mean ± SD (**P < 0.01 versus the sham group; ^##^*P* < 0.01 versus the vehicle group; ^#^*P* < 0.05 versus the vehicle group); scale bars = 25 μm.

To further examine the pyroptosis in spinal cord, we performed double immunofluorescence staining. It was determined as pyroptosis if double positive cleaved Caspase-1 and TUNEL (or PI) occurred. The PI staining measurement revealed that the double positive expressions were up-regulated in the vehicle group compared with the sham group (*P* < 0.001) (Figure 5A). The TUNEL staining measurement gave similar results (*P* < 0.001) (Figure 5B).

These results revealed the presence of activation of NLRP3 inflammasome and pyroptosis in NCLDH models.

### MaR1 Suppressed NLRP3 Inflammasome Activation and Pyroptosis

Whether MaR1 promoted inflammatory resolution and alleviated radicular pain by suppressing the activation of NLRP3 inflammasome and pyroptosis was evaluated in this study. Accordingly, after intrathecal delivery of MaR1 in the vehicle group, mRNA expression levels of inflammasome and pyroptosis-related indicators were first determined via qRT-PCR. The results showed that MaR1 (10 or 100 ng) could significantly attenuate the increased mRNA expression of NLRP3 (*P* < 0.01, *P* < 0.001), ASC (*P* = 0.039, *P* < 0.001), and Caspase-1 (*P* < 0.01, *P* < 0.001), induced by NCLDH, and that of GSDMD (*P* < 0.01, *P* < 0.001) dose-dependently (Figures 3A–D). The intrathecally injected MaR1 (10 or 100 ng) also appeared to significantly reverse the increased expression of NLRP3 (*P* < 0.01, *P* < 0.001), ASC (*P* = 0.023, *P* < 0.01), Caspase-1 p10 (*P* = 0.032, *P* < 0.01), and GSDMD (*P* = 0.033, *P* < 0.01) by Western blot analysis in a dose-dependent manner (Figures 3E–I).

The comparison of the change in positive expressions of GSDMD and NLRP3 in the spinal dorsal horns was performed using immunohistochemistry after delivery of MaR1. The results showed that intrathecal administration of MaR1 (10 or 100 ng) dose-dependently attenuated the NLRP3 activation (*P* = 0.043, *P* < 0.001) and GSDMD activation (*P* = 0.034, *P* = 0.011) (Figures 4A–D). Also, the cell death analysis revealed that MaR1 treatment attenuated pyroptosis dose-dependently according to PI staining (*P* = 0.028, *P* < 0.001) and TUNEL staining (*P* = 0.027, *P* < 0.001) (Figures 5A,B).

**FIGURE 6:**
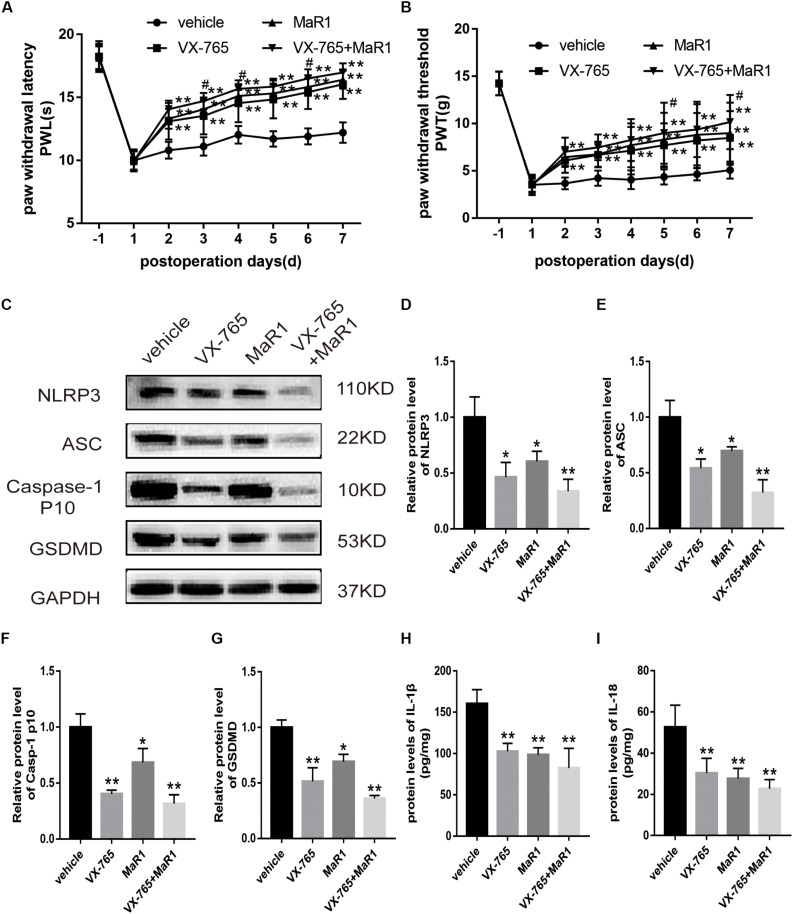
MaR1 attenuated inflammatory response by inhibiting NLRP3 inflammasome activation (*n* = 6/group). The operative rats were injected with Caspase-1 inhibitor (VX-765) intraperitoneally with or without intrathecal administration of MaR1. **(A,B)** The mechanical and thermal hypersensitivity were assessed to evaluate the analgesic effect. **(C–G)** The protein expressions of NLRP3 inflammasome and pyroptosis-related indicators were analyzed by Western blot. **(H,I)** The IL-1β and IL-18 protein levels were tested by ELISA. Data are given as the mean ± SD (**P < 0.01 versus the vehicle group; *P < 0.05 versus the vehicle group; ^#^*P* < 0.05 versus the VX-765 group).

Then, we injected Caspase-1 inhibitor (VX-765) intraperitoneally to the operative rats with or without intrathecal administration of MaR1 to inspect the regulation of MaR1 on inflammasome-mediated pyroptosis. MaR1 and VX-765 alone both improved the mechanical and thermal hypersensitivity, and the combination of them made additional effect (Figures 6A,B). The mechanical allodynia in the co-treatment group was alleviated significantly than that in the VX-765 group on the 5th day (*P* = 0.041) and the 7th day (*P* = 0.045). For the thermal stimulus, the combination of MaR1 and VX-765 attenuated hyperalgesia markedly compared with the VX-765 group from the 3rd day (*P* = 0.033) to the 6th day (*P* = 0.043), except for the 5th day (*P* = 0.136). MaR1 blocked the protein expressions of NLRP3, ASC, and cleaved Caspase-1 induced by NCLDH, and Caspase-1 inhibition further enhanced the decreasing expression levels (Figures 6C–F). Meanwhile, VX-765 treatment decreased the GSDMD expression, and MaR1 injection further decreased the expression (Figure 6G). Then, we evaluated the effect of the levels of inflammatory cytokines. The IL-1β and IL-18 induction was suppressed by Caspase-1 inhibitor, and co-treatment with MaR1 strengthened the down-regulated IL-1β and IL-18 levels (Figures 6H,I).

Therefore, we confirmed that MaR1 attenuated pyroptosis triggered by NCLDH, and NLRP3 inflammasome inactivation induced by Caspase-1 inhibition facilitated this process.

### MaR1 Inhibited Pyroptosis via the NF-κB Pathway

**FIGURE 7:**
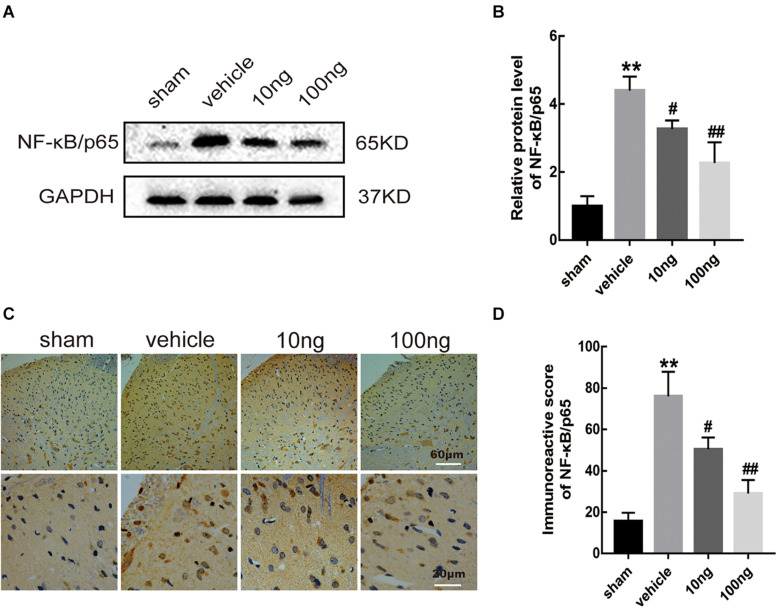
MaR1 reduced levels of protein expression and positive images of NF-κB/p65 (*n* = 6/group). Western blot and immunohistochemistry were used to determine the levels of positive expression of NF-κB/p65 in spinal dorsal horns. **(A,B)** MaR1 (10 or 100 ng) reversed the rising protein expression levels of NF-κB/p65 induced by NCLDH. **(C,D)** The immunostaining images of NF-κB/p65 in the spinal dorsal horns were significantly increased in the vehicle group. Intrathecal delivery of MaR1 (10 or 100 ng) decreased the positive expression of NF-κB/p65. Data are given as the mean ± SD (**P < 0.01 versus the sham group; ^##^*P* < 0.01 versus the vehicle group; ^#^*P* < 0.05 versus the vehicle group).

Similar to our previous study (Liu et al., 2016), the results from Western blot and immunohistochemistry analyses indicated the marked increase in the expression of NF-κB/p65 (*P* < 0.001 for each) in the NCLDH model. According to Western blot, intrathecal administration of MaR1 (10 or 100 ng) reduced the expression level of NF-κB/p65 (*P* = 0.015, *P* < 0.01) (Figures 7A,B). The same results were validated by immunohistochemistry (*P* = 0.028, *P* < 0.01) (Figures 7C,D). Therefore, we conclude that MaR1 alleviated the inflammatory response and inhibited pyroptosis via the NF-κB/p65 pathway.

## Discussion

It has been widely acknowledged that the immune and inflammatory cascades triggered by a protruded NP are solely responsible for the pathogenesis of radicular pain (Geiss et al., 2007; Park et al., 2011; Di Martino et al., 2013). This study indicated that the pyroptosis mediated by NLRP3 inflammasome critically contributed to the pathogenesis of radicular pain. MaR1 could inhibit the NLRP3 inflammasome activation and pyroptosis and thereby effectively promote inflammation resolution and improve signs of pain dose-dependently.

The inflammation resolution is biosynthetically active. During this process, endogenous SPMs play key roles in maintaining host homeostasis (Buckley et al., 2014; Serhan, 2014). As a novel family of DNA-derived pro-resolving lipid mediators, maresins are produced mainly by macrophages (Serhan et al., 2015). As the member identified firstly in this family, MaR1 displays a series of actions to re-establish host homeostasis, such as promoting inflammation resolution, accelerating tissue regeneration, and ameliorating neuropathic pain (Serhan et al., 2012). Increasing evidence has shown that the actions of MaR1 in anti-inflammatory and proinflammatory resolution are more promising than those already reported such as resolvins (Serhan et al., 2009, 2012; Dalli et al., 2013).

In our previous studies, we have proved the up-regulation of proinflammatory cytokines (IL-6, IL-1β, and TNF-α) in our animal model and that intrathecal administration of SPMs could effectively reduce the level of cytokines and alleviate mechanical allodynia by different pathways (Miao et al., 2015; Liu et al., 2016; Zhang et al., 2018). Similarly, in this study, MaR1 promoted the inflammatory response resolution (decreasing the TNF-α, IL-18, and IL-1β levels), thereby improving the pain behavior induced by NCLDH dose-dependently. However, the mechanism of cytokine generation is unclear. Whether MaR1 could inhibit the upstream inflammatory cascades is of great interest to us.

Inflammasomes recognized exogenous and endogenous dangerous signals involved in the process of neuroinflammation (Walsh et al., 2014). A growing number of researches have illustrated the contribution of NLRP3 inflammasome in developing neuropathic pain, e.g., chemotherapy-induced pain (Jia et al., 2017), injury-evoked pain (Pan et al., 2018), and diabetic neuropathic pain (Liu et al., 2017). However, it has been seldom reported whether cell pyroptosis contributes to radicular pain. We provided evidence in our current study that NLRP3 inflammasome activation and cell pyroptosis were associated with radiculopathy in a rat model of NCLDH.

The NLRP3 inflammasome is an important inflammasome complex that can cleave and activate Caspase-1 by directly interacting with ASC, triggering cell pyroptosis (Bergsbaken et al., 2009; Schroder et al., 2009). Our results showed that multiple proteins that were associated with inflammasome (e.g., NLRP3, ASC, and Caspase-1) were highly expressed in the ipsilateral spinal cord. Recent studies determined that GSDMD, with the gasdermin-N domain, was responsible for membrane pore-forming activity and pyroptosis execution (Shi et al., 2015; Sborgi et al., 2016). Therefore, we assumed that the GSDMD protein was essential for MaR1 to inhibit cell pyroptosis in the spinal cord. This study indicated that MaR1 could significantly inhibit the mRNA and protein expression of GSDMD. Also, MaR1 reduced cell death in spinal cord according to PI and TUNEL staining. Therefore, we concluded that MaR1 attenuated NLRP3 inflammasome and pyroptosis to promote inflammatory resolution.

In addition, our study showed that VX-765 injection could suppress the expression of proteins related to NLRP3 inflammasome and pyroptosis and attenuate mechanical and thermal allodynia. In a previous study, Caspase-1 inhibition by VX-765 has been shown to prevent NLRP3 inflammasome activation and pyroptosis (McKenzie et al., 2018). We have further evidence that MaR1 treatment could promote inflammatory resolution, attenuate radicular pain, and prevent NLRP3 inflammasome activation and pyroptosis. Therefore, we deduced that MaR1 relieved radicular pain targeting NLRP3 inflammasome and pyroptosis. However, which factor triggered the activation of NLRP3 inflammasome and pyroptosis in the model needed to be further studied. The reproduction of reactive oxygen species (ROS) is likely responsible for the pathology of intervertebral disc degeneration (Lin et al., 2018). Thus, we supposed that ROS induced by oxidative stress and inflammatory stimulus was the major factor to activate NLRP3 inflammasome. The specific mechanism will be evaluated in the following study.

In the process, we assumed that the antigenic NP tissue that was exposed to the systemic circulation in disc herniation induced the activation of NLRP3 inflammasome accompanied by proinflammatory cytokine secretion and cell pyroptosis. The inflammasome was further activated by the release of cell contents, leading to the inflammatory cascades. Excessive inflammatory and immune responses, which are mediated by inflammasome activation, could probably aggravate the injury of nerve tissue (Lamkanfi and Dixit, 2014; Guo et al., 2015), which induced central sensitization in the spinal cord and the pain syndrome (Moalem and Tracey, 2006; Ji et al., 2011). MaR1 prompted inflammatory resolution and neuroprotection (Francos-Quijorna et al., 2017) through inhibiting the upstream proinflammatory cascades. Similarly, we found that the combination of MaR1 and VX-765 made an additional effect. MaR1 probably mitigated pain symptom via other pathways.

It has been demonstrated that the NF-κB family could act as a crucial contributor in inflammation and immune processes (Barnes and Karin, 1997). By NF-κB signaling, local inflammation could be induced by inflammatory cytokines (IL-1β, IL-18, and TNF-α) (O’Neill and Greene, 1998; Lawrence, 2009). Our previous studies and other researches demonstrated that SPMs (e.g., lipoxin A4, resolvin D1, and Maresin 1) enabled inflammatory resolution by inhibiting NF-κB signaling (Miao et al., 2015; Li et al., 2016; Liu et al., 2016; Yin et al., 2017). Inflammasome-induced pyroptosis was alleviated by inhibiting the NF-κB/GSDMD pathway in mouse adipose tissue (Liu et al., 2017). It has been demonstrated that phosphorylated NF-κB bound upstream of the GSDMD promoter region to elevate GSDMD transcription (Liu et al., 2017). Therefore, we preliminarily determined that through the NF-κB pathway, MaR1 played a key role in inhibiting NLRP3 inflammasome activation and attenuating pyroptosis. Our results also indicated that the NF-κB/p65 protein expression had a significant up-regulation in the vehicle group and a reduction after administration of MaR1, which sufficiently proved our presumption.

In this study, the activation of NLRP3 inflammasome and pyroptosis were found to critically contribute to the radicular pain. Also, we demonstrated that MaR1 could effectively reduce inflammatory response and relieve neuropathic pain by inhibiting pyroptosis via NF-κB signaling. Our study revealed a novel role of MaR1 in inhibiting NLRP3 inflammasome-induced pyroptosis, providing a new potential mechanism for specialized pro-resolving mediators (SPMs) to promote inflammatory resolution and alleviate radicular pain.

## Data Availability Statement

The datasets generated for this study are available on request to the corresponding author.

## Ethics Statement

The animal study was reviewed and approved by the Shandong University Animal Care and Use Committee.

## Author Contributions

YW was responsible for carrying out the major part of the study, the statistical analyses, and writing the manuscript. YL helped in conducting the animal models and Western blot study, and made the major work during the revision. JW carried out the behavioral tests and injected drugs. QZ collected the tissues and carried out the ELISA study. JJ and SWe were in charge of the immunofluorescence study. SWa carried out the real-time polymerase chain reaction. TS conceived and designed the study, and revised the manuscript. All authors contributed to the article and approved the submitted version.

## Conflict of Interest

The authors declare that the research was conducted in the absence of any commercial or financial relationships that could be construed as a potential conflict of interest.

## Supplementary Material

FIGURE S1 Assessment of pain behavior in contralateral side and sham groups with MaR1 (*n* = 8/group). **(A,B)** The contralateral PWL and PWTs didn’t show any differences among the four groups. **(C,D)** The PWL and PWTs were not different in the sham rats with or without MaR1 treatment. Data are presented as the mean ± SD.

FIGURE S2 Comparison of the stability of β-action and GAPDH mRNA expression in spinal dorsal horns (*n* = 5/group). The stability of β-actin expression was confirmed in spinal dorsal horns under several different treatments by comparison of Ct values of β-action and GAPDH.

## Abbreviations

DRG: dorsal root ganglion
GSDMD: gasdermin D
NCLDH: non-compressive lumbar disc herniation
NLRP3: NOD-like receptor (NLR) family pyrin domain-containing protein 3
NP: nucleus pulposus
PWL: paw withdrawal latency
PWTs: paw withdrawal thresholds
ROS: reactive oxygen species.
