# Supplementary figures and images for: Maresin 1 Attenuates Radicular Pain Through the Inhibition of NLRP3 Inflammasome-Induced Pyroptosis via NF-κB Signaling
Source: Front Neurosci. 2020 Aug 26;14:831. doi: 10.3389/fnins.2020.00831 (PMC7479972; doi:10.3389/fnins.2020.00831)

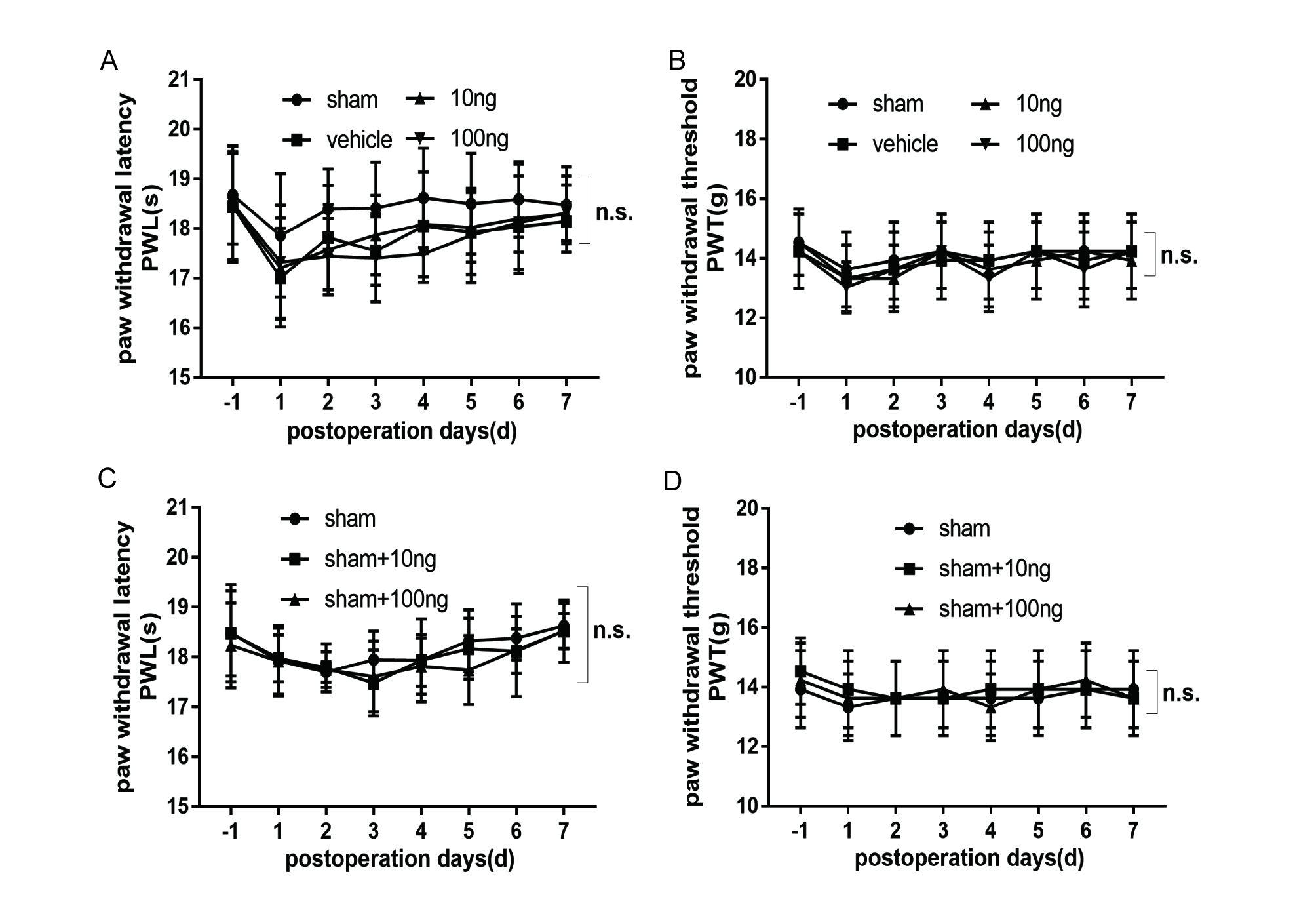

Supplement: FIGURE S1 — Assessment of pain behavior in contralateral side and sham groups with MaR1 (n = 8/group). (A,B) The contralateral PWL and PWTs didn’t show any differences among the four groups. (C,D) The PWL and PWTs were not different in the sham rats with or without MaR1 treatment. Data are presented as the mean ± SD. [file Image_1.tif]

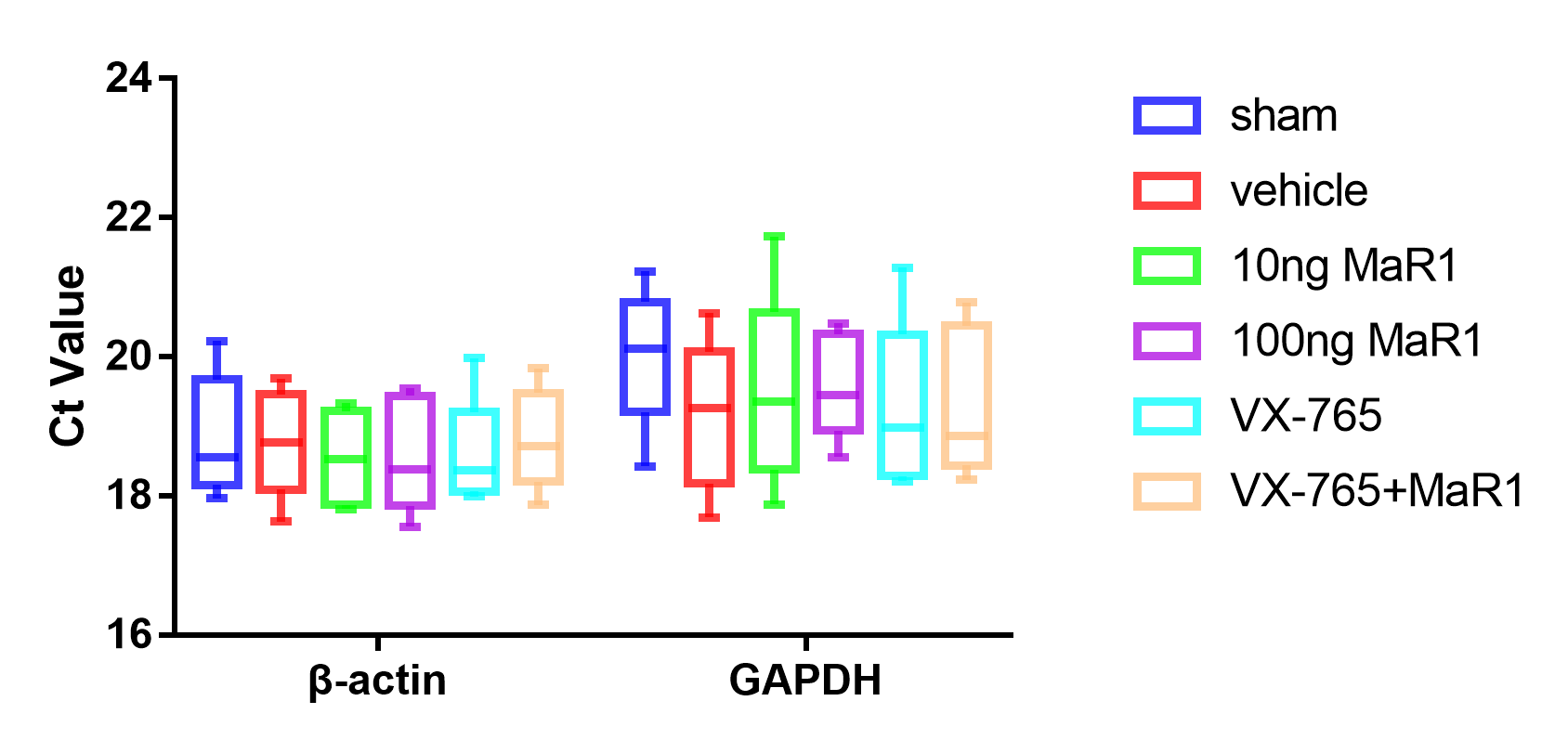

Supplement: FIGURE S2 — Comparison of the stability of β-action and GAPDH mRNA expression in spinal dorsal horns (n = 5/group). The stability of β-actin expression was confirmed in spinal dorsal horns under several different treatments by comparison of Ct values of β-action and GAPDH. [file Image_2.tif]
